# Computed tomography (CT) scan challenges the result of SARS-CoV-2 nucleic acid test in a suspected COVID-19 case

**DOI:** 10.1017/ice.2020.171

**Published:** 2020-04-22

**Authors:** Kun Yan, Jingfeng Zhang, Yangfan Zhang, Shun Zhang, Ting Cai, Jianjun Zheng

**Affiliations:** 1 Hwa Mei Hospital, University of Chinese Academy of Sciences, Ningbo, Zhejiang, China; 2 Ningbo Institute of Life and Health, University of Chinese Academy of Sciences, Ningbo, Zhejiang, China; 3 Ningbo University School of Medicine, Ningbo, Zhejiang, China

*To the Editor*—At present, COVID-19 is a global pandemic. The reverse-transcriptase polymerase chain reaction (RT-PCR) test is the gold standard for diagnosing COVID-19. We report a case with the false-positive result of SARS-CoV-2 nucleic acid testing in which chest computed tomography (CT) findings challenged the diagnosis of COVID-19 pneumonia.^1,2^

**Fig. 1.**
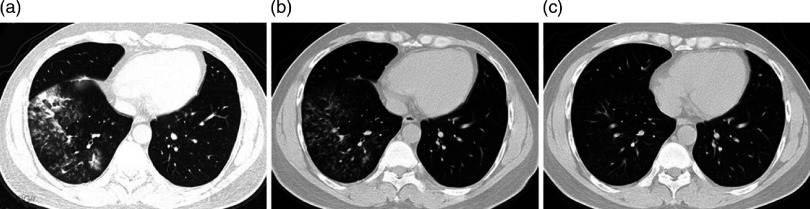
Chest computed tomography (CT) images of a 36-year-old man with a false-positive nucleic acid test result for SARS-CoV-2. The revised diagnosis was community-acquired pneumonia. Panel A: chest CT obtained on February 16 shows large, mixed, ground-glass opacity (GGO) in the right lower lobe with partial consolidation, distribution along with bronchovascular bundles, and a little effusion of the right oblique fissure. Panel B: a CT image obtained on February 21 demonstrates that the lesions in the right lower lobe have been partially absorbed. Panel C: a CT image obtained on February 29 shows that the lesions in the lower right lobe have completely resolved.

On February 16, 2020, a 36-year-old man presented to a local primary healthcare clinic with a mild cough and subjective fever for 1 day. He had no history of contact with COVID-19 patients. Physical examination revealed a body temperature of 38.4°C. A routine blood panel showed normal ranges of white blood cells count (8.9×10^9^/L), neutrophils (6.1×10^9^/L), and lymphocytes (1.8×10^9^/L), as well as a high level of C reactive protein (25.2 mg/L). An oropharyngeal swab tested negative for influenza A and B but positive for SAR-CoV-2 by RT-PCR on February 17. The patient’s chest CT showed large, mixed, ground-glass opacity (GGO) in the lower lobe of right lung with partial consolidation, distribution along with bronchovascular bundles, and a little effusion of the right oblique fissure (Fig. 1, Panel A).

On February 18, the patient was transferred to our hospital for isolation and treatment as a “confirmed case.” According to a consultation among a multidisciplinary team, the diagnosis for COVID-19 seemed to be questionable according to the CT manifestations. Therefore, some imperative measures were taken as follows: (1) the patient was isolated in a single ward; (2) the same specimen was recollected as soon as possible and testing was repeated; (3) the patient’s serum was tested for the virus-specific antibody of IgM for SAR-CoV-2; (4) a blood culture for bacteria or fungi was performed; and (5) an antibacterial agent was administered (moxifloxacin hydrochloride tablets, 0. 4 g orally 4 times per day) as well as an antiviral agent (lopinavir/ritonavir tablets 400 mg/100 mg orally twice daily).

The patient’s serum tested negative for SARS-CoV-2–specific IgM antibody on February 18. Consecutive samples were collected for SARS-CoV-2 testing daily from February 18 to February 20 (ie, oropharyngeal swab and sputum). All of the repeated tests of viral nucleic acid were negative. A 5-day blood culture demonstrated no growth of bacteria or fungi. The patient’s symptoms gradually improved. A repeat chest CT scan on February 21 showed that the lesions in the right lower lobe had been partially absorbed (Fig. 1, panel B).

On February 22, bronchoalveolar lavage fluid was collected for SARS-CoV-2 testing by RT-PCR and the result was also negative. On February 24, the patient’s serum tested negative for SARS-CoV-2–specific IgG antibody. All of his symptoms had disappeared by February 26. A follow-up chest CT on February 29 showed that the lesions in the right lower lobe had completely resolved (Fig. 1, panel C). After a consultation with the COVID-19 prevention and control committee in our hospital, the diagnosis was revised to community-acquired pneumonia (CAP) considering no history of contact with COVID-19 patients and the negative results of consecutive repeat RT-PCR tests and the serum antibody test. The patient was released from quarantine and was discharged on the same day.

Yan et al^3^ reported that failing to consider COVID-19 because of a positive rapid test result for dengue fever, which has serious implications not only for the patient but also for public health. Our case highlights the importance of timely recognition of a false-positive result for SARS-CoV-2 when chest CT findings do not conform to the typical changes of COVID-19 pneumonia. From the epidemic control perspective, it is imperative to isolate individuals with suspected cases of COVID-19 to protect the uninfected population. However, the suspected cases should not be housed with confirmed cases of COVID-19 to prevent the spread of this disease.
